# Research progress of Chinese medicinal monomers in the process of melanoma occurrence

**DOI:** 10.1080/13880209.2024.2445695

**Published:** 2025-01-10

**Authors:** Yan Shang, Hailong Zhao

**Affiliations:** Department of Pathophysiology, Zunyi Medical University, Zunyi, China

**Keywords:** Melanoma, Chinese medicinal monomers, herbal antitumor activity, traditional Chinese medication (TCM), melanomagenesis inhibition

## Abstract

**Context:**

Melanoma’s aggressiveness and resistance to radiotherapy highlight an urgent need for innovative treatments. Traditional Chinese medicine (TCM) offers a unique approach through its ‘four natures’ theory—cold, cool, warm, and hot.

**Objective:**

This review aims to explore the potential of TCM’s ‘four natures’ herbal monomers in melanoma treatment, providing an alternative to conventional therapies.

**Materials & methods:**

A systematic literature review was conducted by accessing various databases, including Baidu Scholar, PubMed, Science Citation Index Expanded (SCIE), and China National Knowledge Infrastructure (CNKI), to synthesize the most recent findings on traditional Chinese medicine monomers. Furthermore, this review elucidated the mechanisms underlying their role in melanoma retention.

**Results:**

TCM’s multi-component, multi-target approach has shown promise in addressing melanoma’s complexity, with specific monomers demonstrating the ability to modulate tumor behavior.

**Discussion and Conclusions:**

The ‘four natures’ theory in TCM presents a novel perspective for melanoma treatment, warranting further investigation into its clinical applications and potential integration with modern oncology.

Melanoma is a highly aggressive cancer, prone to metastasis, and primarily affects the skin and mucous membranes. According to the American Cancer Society’s 2023 statistics on cancer outcomes, the incidence of melanoma, along with liver, breast, and uterine body cancers, is consistently increasing each year (Siegel et al. 2023). Although there are numerous treatment options available for melanoma today, mainly including surgical resection, targeted and immunotherapy, and chemotherapy and radiotherapy, a lack of awareness about melanoma makes it difficult to detect in its early stages (Mao et al. 2020). Furthermore, patients with advanced melanoma not only face treatment challenges and low response rates but are also prone to tumor drug resistance.

The theory of the Four Natures in traditional Chinese medicine (TCM) is not only an integral part of the broader TCM theoretical framework but also the core of the pharmacological properties of Chinese herbal medicine. The Four Natures mainly include cold, cool, warm, and hot, which represent the corresponding reactions that occur within the body after the administration of Chinese herbs. This is a distinctive attribute unique to Chinese herbal medicine (Wang et al. 2023). Chinese herbal monomers with the Four Natures exhibit a comprehensive anti-tumor effect, targeting multiple levels and pathways within the body. They are known for their efficacy and safety when used in accordance with traditional practices. Furthermore, monomers from Chinese medicinal herbs, characterized by the Four Natures, have been extensively utilized in the treatment of various tumors. This includes a range of natural products and their derivatives, such as agaric (if ‘acacia ear’ refers to a type of mushroom), heavy floor (which may need clarification or correction), paclitaxel alkaloids, and essential oils (Xiao et al. 2012). Therefore, Chinese medicine with the Four Natures can offer a feasible method for researching the treatment of tumors. The objective of this paper is to elucidate the effects and underlying mechanisms of Four-Nature herbal monomers on melanoma, and to review the advancements in their application to melanomagenesis research (Figure 1). Furthermore, in this systematic review, we intend to critically assess the therapeutic efficacy of traditional Chinese medicine (TCM) monomers in melanoma treatment.

To uphold the methodological rigor and pertinence of the evidence base, we have delineated stringent inclusion and exclusion criteria. The inclusion criteria encompass randomized controlled trials (RCTs), cohort studies, case-control studies, and both prospective and retrospective analyses that investigate the application of TCM monomers for melanoma treatment, with participants being patients with confirmed melanoma diagnoses. We have limited our scope to peer-reviewed articles published in Chinese or English over the preceding decade. The exclusion criteria dismiss studies that are irrelevant, duplicates, have incomplete data, feature single-arm interventions without comparators, include animal or *in vitro* studies, and exclude non-empirical research. These criteria ensure that the synthesized research is both representative and scientifically robust, thereby offering novel insights and potential therapeutic strategies for melanoma management.

**Figure 1.**
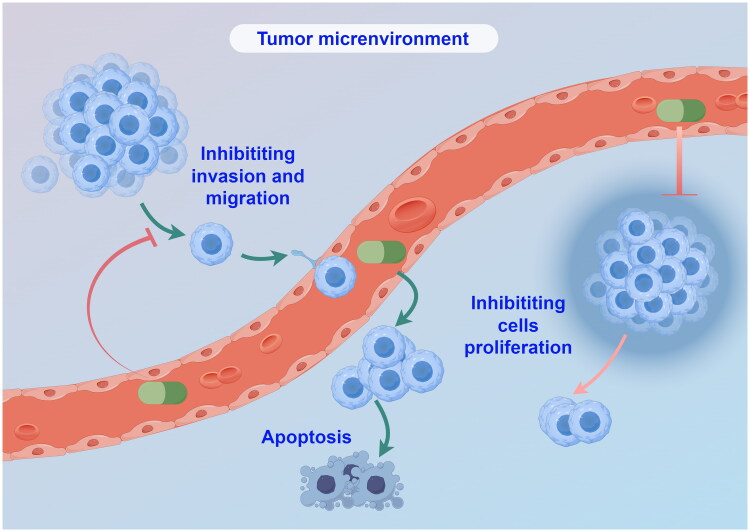
Mechanism diagram of the main actions of Chinese medicinal monomers (by Figdraw).

## Cold-natured Chinese medicinal monomers

**Figure 2.**
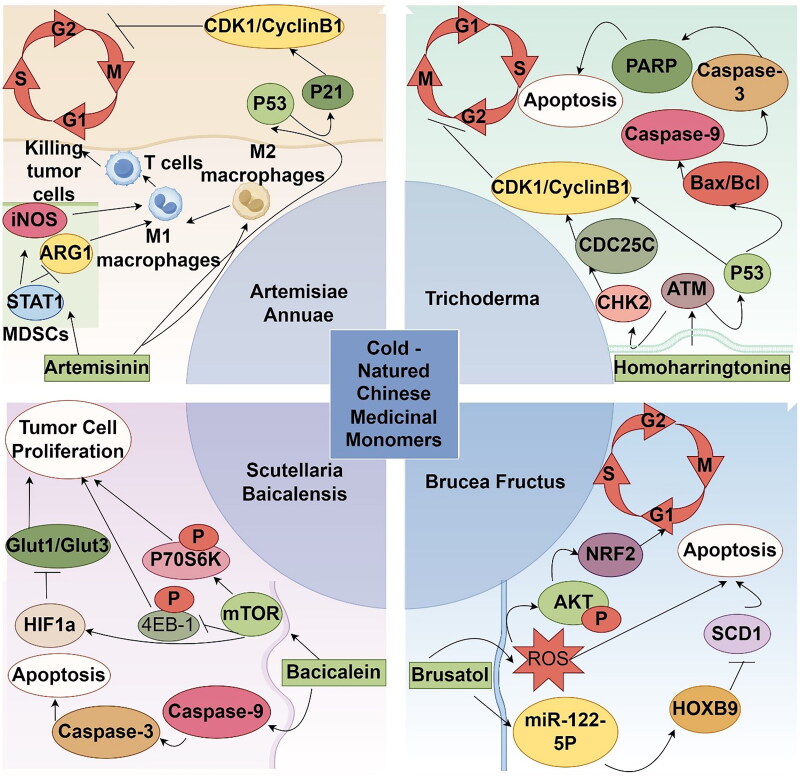
Mechanism diagram of the main actions of cold-natured Chinese medicinal monomers in melanoma (by Figdraw).

Cold herbs typically have a bitter taste and are associated with the Heart, Lung, Liver, Kidney, and Triple Burner meridians in TCM. They are known for their effects on dispersing wind-heat, clearing heat to treat diarrhea, cooling the blood, and detoxifying toxins. Monomers derived from cold herbal sources exhibit a range of pharmacological effects, including antioxidant, anti-inflammatory, and antimicrobial properties. They also have significant antitumor roles, particularly in melanoma and other cancers. Examples of such herbs include Trichoderma, Bruceae fructus, Scutellaria, and Artemisia (Figure 2).

### Monomers of TCM of trichoderma origin

The Trichoderma plant, known for its cold nature and bitter, astringent taste, contains the small molecule alkaloid homoharringtonine (HHT), which is extracted from its branches and leaves. HHT possesses a range of pharmacological effects, including the inhibition of protein and DNA synthesis in tumor cells, the induction of tumor cell apoptosis, and the suppression of tumor cell proliferation (Wang et al. 2021).

It has been found that HHT regulates the levels of proteins such as ATM, p53, γ-H2AX, PARP, Caspase-3, Caspase-9, Bcl-2, Bax, Aurka, Plk1, Cdc25c, CDK1, CyclinB1, and Myt1. *In vitro*, HHT induces apoptosis in melanoma A375 cells. Additionally, the mechanism by which HHT induces cell cycle arrest in the G2/M phase may involve the Aurka/Plk1/Cdc25c and ATM/Chk2/Cdc25c signaling pathways (Tang et al. 2021). This study highlights the therapeutic potential of traditional Chinese medicine-derived monomers in modulating cell cycle progression and inducing apoptosis, thereby contributing to the advancement of modern oncology. In the xenograft model, tumor growth in the HHT treatment group was significantly suppressed. Histological examination (HE staining) revealed pronounced tumor necrosis, characterized by nuclear pyknosis and karyorrhexis, with a scarcity of viable cells. Furthermore, throughout the treatment period, there was no significant alteration in the body weight of mice in the HHT treatment group, suggesting minimal side effects of the drug on the animals (Tang et al. 2021).

It has been reported that HHT enhances the sensitivity of A375R to vemurafenib in BRAF-mutant melanoma cells that have become resistant, specifically the A375R cells. HHT not only achieves this by acting on these resistant cells but also effectively inhibits their developmental process. The mechanism of action may involve the inhibition of the IRS4/PI3K/Akt and Erk signaling pathways, which could play a role in reversing the drug resistance of tumor cells (Huang et al. 2021).

### Monomers of TCM of Bruceae fructus origin

Bruceae fructus, recognized in TCM as cold in nature and bitter in taste, is associated with the liver and large intestine meridians and is slightly toxic. Brusatol, a lactone compound extracted from Bruceae fructus, is known for its ability to inhibit tumor cell proliferation and induce apoptosis.

Brusatol serves as a specific inhibitor of the nuclear factor erythroid 2-related factor (*NRF2*), enhancing the treatment sensitivity of drug-resistant tumors. Furthermore, it exhibits anti-tumor activity by modulating *NRF2* expression. *NRF2* is strongly associated with the development of drug resistance in melanoma. It serves as a key transcriptional target for redox responses in melanoma cells, and its pathway can be strongly activated to protect tumor cells from oxidative stress (Carpenter et al. 2022). In addition to this, *NRF2* can exhibit significant nuclear accumulation in melanoma A375 cells, especially in drug-resistant melanomas (Khamari et al. 2018). Not only that, *NRF2* can facilitate tumor cell immune evasion by inducing the metabolism and efflux of chemotherapeutic agents, thereby potentially undermining the effectiveness of immunotherapies (Harder et al. 2017). UVA treatment of melanoma cells was found to upregulate the expression of the NRF2 protein, however, brusatol reduced NRF2 protein levels in a concentration-dependent manner (Wang et al. 2018). This underscores the significant role of traditional Chinese medicine-derived monomers in modulating the oxidative stress response and dampening immune evasion, both of which are pivotal mechanisms in contemporary oncological research. Co-treatment with brusatol and UVA led to diminished expression of CyclinD1, CyclinE2, CDK4, and CDK6, culminating in G1 phase cell cycle arrest. This intervention effectively inhibited A375 cell proliferation and induced apoptosis (Wang et al. 2018). (Wang et al. 2018) demonstrated that exposure to UVA radiation enhanced the levels of reactive oxygen species (ROS) induced by brusatol and modulated cell survival and cell cycle regulatory proteins. Additionally, (Ding et al. 2018) confirmed that brusatol not only suppressed the proliferation of melanoma A375 cells but also triggered ROS production and apoptosis.

In addition to this, brusatol significantly down-regulates the expression of stearoyl coenzyme A desaturase 1 (SCD1), thereby exerting an anti-melanoma effect. Additionally, miR-122-5p can act as an anti-melanoma cofactor of brusatol, reducing the expression of the transcription factor *HOXB9*. This reduction decreases *HOXB9*’s binding to the SCD1 promoter, enhancing the anti-melanoma effect. The mechanism of action described above may be mediated through the miR-122-5p/*HOXB9*/SCD1 signaling axis (Guo et al. 2023). Moreover, in a xenogeneic animal model, the administration of brusatol resulted in a significant decrease in melanoma tumor volume and weight, along with a reduction in the drug’s toxic side effects. This was achieved not only through the observed tumor reduction but also by lowering the dosage of brusatol to one-third of the original amount (Guo et al. 2023; Li et al. 2023).

### Monomers of TCM of Scutellaria baicalensis origin

Scutellaria baicalensis, a member of the Labiatae family and commonly known as Baikal skullcap, is characterized as cold in nature and bitter in flavor within TCM. It is associated with the lung, gallbladder, spleen, and intestinal meridians. The plant is recognized for its pharmacological properties, which include heat-clearing, detoxification, anti-inflammatory, antibacterial, antioxidant, antitumor activities, and neuroprotective effects (Huang et al. 2024).

The monomers baicalein and wogonin, derived from Scutellaria baicalensis, can promote the polarization of macrophages in the tumor microenvironment from the M2 type to the M1 type. This polarization leads to the secretion of a variety of antitumor factors by M1-type macrophages, such as TNF-α, IL-1β, CXCL9, and CXCL10, which play an antitumor role (He et al. 2021; Zhang et al. 2021). The mechanism of action may involve the JAK2-STAT1 signaling pathway and the NF-κB/TNF-α signaling pathway. Furthermore, baicalein not only influences macrophages in the tumor microenvironment but also enhances the activity of CD4+ and CD8+ T cells. It stimulates the secretion of cytokines, such as IL-2, TNF-α, and IFN-γ, thereby exerting an anti-melanoma effect. Baicalin enhances the secretion of tumor-inhibitory cytokines by immune cells within the tumor microenvironment, aligning with contemporary immunomodulatory strategies in oncology. This property suggests that baicalein could serve as a potential targeted therapeutic agent and an adjuvant for tumor cell vaccines in antimelanoma strategies (Liu et al. 2018).

Furthermore, baicalein has potential as a therapeutic agent against *BRAF*-mutant melanoma. For instance, (Huang et al. 2020) demonstrated that baicalein significantly inhibited the proliferation of A375 cells and reduced the formation and migration of tumor cell colonies. It also increased the expression of Cleaved-Caspase-3, enhancing apoptosis. Baicalein significantly reduced the expression of glucose transport proteins Glut1 and Glut3, as well as key glycolytic enzymes, thereby inhibiting cellular glycolysis. The mechanism of action may involve regulation of glucose metabolism through the mTOR-HIF-1α signaling pathway. Furthermore, baicalein has been shown to significantly suppress melanoma proliferation in a xenogeneic animal model. Additionally, it downregulates the expression of glucose transporters GLUT1 and GLUT3, along with key glycolytic enzymes, suggesting an inhibitory effect on melanoma cell glycolysis. Notably, when exerting its action on tumors, baicalein demonstrates minimal toxic side effects on the host organism (Huang et al. 2020; Lei et al. 2024).

### Monomers of TCM of Artemisia annua origin

Artemisia annua, recognized as cold in nature and bitter and pungent in taste in TCM, is associated with the liver and gallbladder meridians. From this plant, the antimalarial drug artemisinin has been discovered. Artemisinin exhibits a range of pharmacological effects, including anti-inflammatory, antimicrobial, antiparasitic, antiviral, and antitumor activities (Guo et al. 2023).

Artemisinin, a sesquiterpene lactone with diverse pharmacological properties, perturbs mitochondrial homeostasis in neoplastic cells and modulates the secretion and functionality of angiogenic factors *via* autocrine and paracrine signaling pathways, thereby inhibiting tumor-associated angiogenesis and preventing the epithelial-mesenchymal transition (EMT) of tumor cells into endothelial-like cells. (Tsui et al. 2019). Furthermore, dihydroartemisinin markedly suppresses the angiogenic capacity of melanoma cells, disrupts their tube-like structures, and inhibits angiogenesis through the downregulation of the PI3K/AKT/mTOR/HIF-1α/VEGF signaling cascade, consequently impeding melanoma metastasis. (Li et al. 2023). A subsequent study demonstrated that dihydroartemisinin not only possesses antimalarial activity but also dose-dependently suppresses melanoma cell invasion, migration, and proliferation. Additionally, the study elucidated that dihydroartemisinin modulates the differentiation and expansion of immune cells within the tumor microenvironment, including CD8+ cytotoxic T lymphocytes (CTLs), CD4+ T helper cells, and regulatory T cells (Tregs), thereby augmenting the antitumor immune response in mice. Furthermore, dihydroartemisinin significantly induces mitochondrial-mediated apoptosis and impedes melanoma progression through the modulation of the STAT3 signaling pathway, suggesting that dihydroartemisinin may serve as a potential alternative therapeutic agent for melanoma treatment. (Yu et al. 2020). Not only that, dihydroartemisinin, a derivative of artemisinin, has demonstrated anti-melanoma properties. Studies have shown that it can inhibit melanoma angiogenesis by modulating the HIF-1α/VEGF/PI3K/Akt signaling pathway. Additionally, dihydroartemisinin has exhibited inhibitory effects on melanoma cell proliferation and metastasis both *in vitro* and *in vivo* (Zhang et al. 2021; Li et al. 2023).

In addition to this, the development of myeloid-derived suppressor cells (MDSCs) plays a significant role in the emergence of resistance to immune checkpoint blockade therapies, such as anti-PD-L1 antibodies, in cancer treatment. Artemisinin has been found to promote the conversion of macrophages from the M2-type to the M1-type, enhancing the anti-tumor effects of M1-type macrophages. This conversion is achieved by up-regulating nitric oxide synthase (iNOS) and down-regulating arginase 1 (ARG1) within myeloid-derived suppressor cells (MDSCs), in a manner independent of statin1 phosphorylation. Furthermore, the mechanism underlying this conversion is believed to involve modulation through the PI3K/AKT, mTOR, and MAPK signaling pathways. Furthermore, artemisinin can contribute to the reversal of drug-resistant melanoma by enhancing PD-L1 immunotherapy efficacy and promoting the infiltration and proliferation of anti-tumor T cells (Zhang et al. 2022).

In conclusion, herbal monomers with cold properties may influence melanoma development *via* several mechanisms, such as regulating the cell cycle, inducing apoptosis, and modulating the tumor microenvironment. These actions offer new insights and potential therapeutic strategies for melanoma treatment. Although these agents exhibit promising therapeutic effects and lower toxicity profiles in preclinical models, further research and clinical validation are essential to ensure their safety and efficacy in human subjects.

## Cool-natured Chinese medicinal monomers

**Figure 3.**
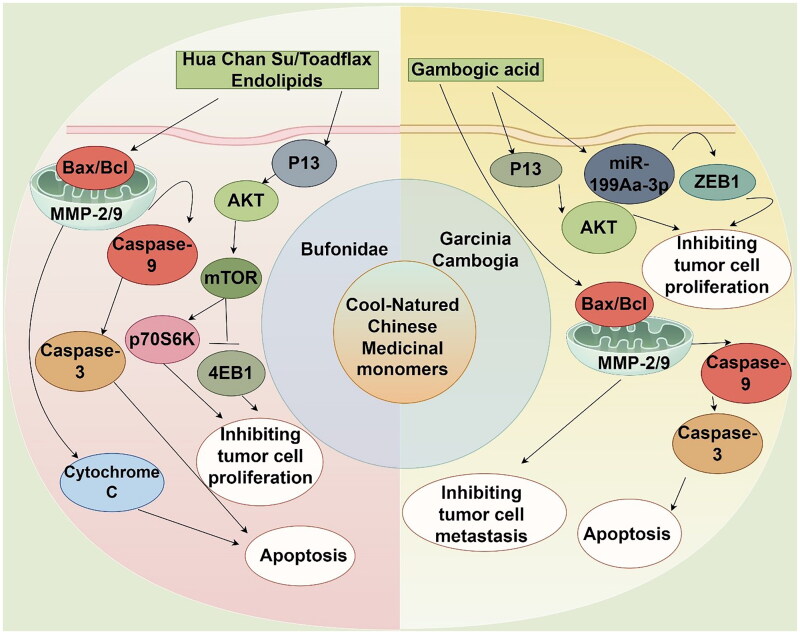
Mechanism diagram of the main actions of cool-natured Chinese medicinal monomers in melanoma (by Figdraw).

Cool-natured Chinese medicinal herbs typically have a sweet and bitter taste, are primarily associated with the heart, lung, liver, and stomach meridians, and possess effects such as clearing heat and detoxifying, promoting blood circulation to remove blood stasis and alleviate pain, as well as benefiting the stomach and generating body fluids. Monomers derived from cool-natured Chinese medicinal sources, both plant-based and animal-based, not only exhibit pharmacological actions such as anti-inflammatory, antimicrobial, and antiviral properties, but also play a significant role in antitumor activities, particularly in melanoma, such as Garcinia cambogia and Bufonidae (Figure 3).

### Monomers of TCM of Garcinia cambogia origin

Garcinia cambogia, recognized as cool in nature and sour and astringent in taste in TCM, is associated with the Stomach and Large Intestine meridians. Garcinia cambogia contains the active chemical component gambogic acid, which exhibits a range of pharmacological effects including organ protection, anti-inflammatory activity, and anti-tumor properties (Sun et al. 2024).

Gambogic acid has been reported to reduce the viability of A375 cells, induce apoptosis, attenuate cell migration and invasion, and increase the sensitivity of drug-resistant cells to therapy. The mechanism underlying these effects may involve the up-regulation of miR-199a-3p, which exerts anti-tumor activity. Additionally, the down-regulation of *ZEB1*, a direct target of miR-199a-3p, may attenuate the resistance of A375 cells to radiochemotherapy (Liang et al. 2018). Study have demonstrated that gambogic acid exhibits anti-melanoma effects by inhibiting epithelial-mesenchymal transition and tumor angiogenesis. It also decreases the activities of MMP-2 and MMP-9 enzymes, thereby reducing the invasion and migration of melanoma cells. Additionally, gambogic acid significantly inhibits the growth of A375 cells. This inhibitory effect may be attributed to its impact on the PI3K/Akt and ERK signaling pathways, which are crucial in the regulation of cell survival and proliferation (Li et al. 2019).

Furthermore, Garcinia cambogia has been associated with certain toxic side effects in clinical use (Zhao et al. 2010). However, research by Zhang et al. (2021) has demonstrated that the local dermal injection of Garcinia cambogia acid is not only efficacious in treating melanoma but also mitigates its toxic effects on various organs. Importantly, this local administration approach has proven to be non-toxic to the skin (Zhang et al. 2021).

### Monomers of TCM of Bufonidae origin

Bufonidae, characterized as cool in nature, pungent, and toxic, is associated with the Heart, Liver, Spleen, and Lung meridians in TCM. The natural active components found in the toad’s venom, particularly from the skin and parotid glands, possess a range of pharmacological effects, including analgesic, hypotensive, antimicrobial, and antitumor activities.

According to (Li et al. 2019), toadflax endolipids, when acting on human malignant melanoma A375 cells, modulate the AKT signaling pathway and its downstream proteins, such as mTOR, p70S6K, GSK-3β, and CyclinD1. By inhibiting this pathway, toadflax endolipids may reverse drug resistance in melanoma. This effect significantly inhibits the proliferation of A375 cells and induces apoptosis. Toadflax endolipids, secreted by Bufonidae, exhibit significant inhibitory effects on melanoma cell lines, including the SK-MEL-1 cell line. While these endolipids are potent, their action in the context of melanoma is therapeutic, highlighting their potential as antitumor agents (Zhou et al. 2021). (Soumoy et al. 2024) discovered that toadarin, a derivative of toadflax, exhibits potent antitumor activity against melanoma. The mechanism underlying this effect appears to involve the up-regulation of *ATP1A1* expression in drug-resistant cells, enhancing their sensitivity to drug treatments. Consequently, toadflax derivatives have the potential to reverse drug resistance in melanoma, offering a promising avenue for further research and development.

Furthermore, Hua Chan Su, a herbal ingredient derived from the dried skin secretions of toads in the Bufonidae family, has demonstrated significant antitumor efficacy against malignant melanoma and the potential to reverse drug resistance in tumor cells. (Pan et al. 2019) discovered that Hua Chan Su could suppress the expression of PI3K, AKT, and Bcl-2, and concurrently increase the levels of Bcl-2-related death promoter, cytosolic cytochrome C, and apoptotic protease activator. These changes resulted in elevated expression of Caspase-9 and Caspase-3, triggering apoptosis. Consequently, Hua Chan Su reduced the drug resistance of A375 cells. The mechanism of action is likely associated with the PI3K/AKT signaling pathway.

In summary, cool-natured Chinese medicinals, including plant- and animal-derived monomers, may combat melanoma through multiple pathways, such as directly inhibiting the growth of melanoma cells, inducing apoptosis in melanoma cells, and regulating the tumor microenvironment. Although these cool-natured herbal monomers have shown potential antitumor effects in *in vitro* experiments, their clinical application still requires validation through further studies.

## Warm-natured Chinese medicinal monomers

**Figure 4.**
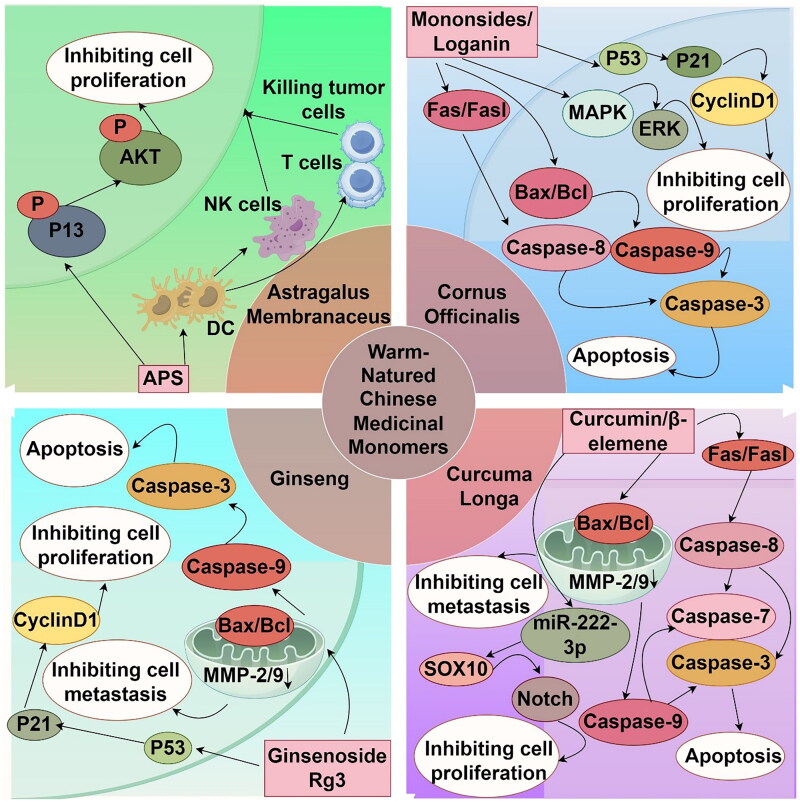
Mechanism diagram of the main actions of warm-natured Chinese medicinal monomers in melanoma (by Figdraw).

Warm herbs, characterized by their pungent and sweet flavors, are primarily associated with the lung, stomach, and spleen meridians in TCM. They serve to disperse surface cold, warm the middle burner, relieve pain, and expel wind and dampness. Monomeric compounds derived from herbs such as Cornus officinalis, Curcuma longa, Ginseng, and Astragalus membranaceus exhibit a range of pharmacological properties, including antioxidant, anti-aging, and immunomodulatory activities. Moreover, they demonstrate antitumor effects, with specific activity against melanoma (Figure 4).

### Monomers of TCM of Cornus officinalis origin

Cornus officinalis, recognized as mildly warm in nature and sour and astringent in flavor, is associated with the Liver and Kidney meridians inTCM. Its main chemical components are cyclic terpene glycosides, including mononosides and loganin, which are known for their immunomodulatory, anti-inflammatory, and antitumor effects. The resistance of melanoma cells, such as the human malignant melanoma A375 cells, to chemotherapeutic drugs has unfortunately led to a low survival rate among patients. Consequently, research into herbal monomers derived from Cornus officinalis may yield potential drugs capable of combating drug-resistant melanoma.

It has been found that mononosides significantly inhibit the proliferation of A375 cells in a dose-dependent manner. Mononosides not only down-regulate Cyclin D1 and up-regulate P21 protein expression to exert anti-tumor effects but also influence the expression of Bcl-2 and Bax at both the protein and gene levels, thereby inducing apoptosis in A375 cells. The mechanism of action is likely related to the activation of the ERK/MAPK signaling pathway (Li et al. 2016). Another study has indicated that loganin suppresses the proliferation of A375 cells in a time- and dose-dependent manner and induces apoptosis by modulating the protein expression levels of Bax, Caspase-3, Fas, and FasL. The mechanism of action is potentially linked to the regulation of the ERK1/2 signaling pathway by its upstream factors, Ras and c-Raf (Dang et al. 2016).

### Monomers of TCM of Curcuma longa origin

Curcuma longa, recognized as warm in nature and bitter and pungent in flavor within TCM, is associated with the Spleen and Liver meridians. Curcuma longa, valued as a TCM in China, exhibits pharmacological activities that extend beyond the traditional theories of ‘activating blood circulation to relieve qi stagnation, regulate menstruation, and alleviate pain.’ Its diverse range of effects has made it a new focal point in the study of natural medicinal compounds. Curcuma longa, also known as turmeric, and its natural extracts exhibit a range of biological activities, including anti-inflammatory, antioxidant, antibacterial, and antitumor properties.

β-Elemene, a terpene compound isolated from Curcuma longa, exhibits significant antitumor activity. It demonstrates a direct cytotoxic effect on tumor cells and also inhibits their proliferation and induces apoptosis. This multifaceted action is mediated through the regulation of various signaling pathways, contributing to its overall antitumor efficacy (Xie et al. 2022). Beyond its direct cytotoxicity, β-elemene has the capacity to reverse drug resistance in tumor cells. For instance, research has shown that β-elemene can counteract resistance to 5-fluorouracil in P53-deficient HCT116p53-/- cells. This is achieved by inducing autophagy and CyclinD3-dependent cell cycle arrest, which enhances the sensitivity of the tumor cells to the chemotherapeutic agent. Furthermore, β-elemene can modulate the expression of miR-1323, thereby inhibiting the metastasis of drug-resistant tumor cells. The underlying mechanism is believed to involve the miR-1323/CbI-b/EGFR signaling axis, playing a role in reversing tumor resistance (Deng et al. 2020; Zhang et al. 2020). Furthermore, while there are no representative clinical studies exploring the early control of melanoma with β-elemene, preliminary research indicates its potential efficacy. β-Elemene has been shown to inhibit the proliferation of A375 cells and activate caspases, including Caspase-3, Caspase-7, and Caspase-9. It also increases the Bax to Bcl-2 ratio, thereby inducing apoptosis *in vitro.* Additionally, β-elemene enhances the sensitivity of these cells to radiotherapy and reduces the likelihood of drug resistance development. The underlying mechanism may involve the regulation of the Fas/FasL expression and the PI3K/Akt/mTOR/p70S6K1 signaling pathway (Balavandi et al. 2020). An additional study has reported that β-elemene, when administered for the treatment of xenograft animal models, does not induce ocular toxicity in mice. Furthermore, β-elemene has been observed to exert no significant impact on the body weight or behavioral patterns of the mice, suggesting an absence of notable systemic toxic side effects (Shi et al. 2015).

Furthermore, LXX-8250, an isopropanolamine derivative of β-elemene, has demonstrated greater potency in tumor cytotoxicity compared to its parent compound. One reported mechanism behind melanoma’s resistance to BRAF or MEK inhibitors involves the upregulation of the autophagic response in tumor cells, allowing them to adapt to the drug pressure (Martin et al. 2017; Mulcahy Levy et al. 2017; Li et al. 2019). LXX-8250 has been shown to inhibit autophagosomal degradation in drug-resistant melanoma cells, thereby inducing apoptosis and reversing the drug-resistant phenotype. This effect on tumor cells counteracts resistance to therapy, suggesting a potential strategy for overcoming treatment resistance in melanoma. Furthermore, LXX-8250 has been shown to down-regulate the expression of PFKFB4, an enzyme related to aerobic glycolysis. This reduction in PFKFB4 expression decreases the production of lactate and fructose-1,6-bisphosphate, which are often overproduced in melanoma cells. Consequently, this inhibition of glycolytic enzymes contributes to the suppression of cellular drug resistance development (Jalal et al. 2022).

Curcumin, a fat-soluble polyphenolic pigment, is extracted from Curcuma longa, which is a TCM plant. Curcumin and its derivatives are widely used in the treatment of various tumors. Their mechanism of action involves inhibiting tumor cell proliferation, invasion, and metastasis by modulating multiple signaling pathways. For instance, they regulate the TLR4/mTOR signaling pathway and influence the Wnt/β-catenin signaling pathway by affecting the expression of Bcl-2-related transcription factors (Deng et al. 2023; Shelash Al-Hawary et al. 2023). Curcumin and its derivatives exert a range of inhibitory effects on melanoma cells, including the suppression of proliferation, invasion, and migration. These effects are mediated through various signaling pathways and also contribute to the reversal of drug resistance in melanoma. (Tang and Cao 2022) discovered that curcumin can reduce SOX10 gene expression by upregulating the expression level of miRNA-222-3p. This upregulation leads to the inactivation of the Notch signaling pathway, which subsequently inhibits the proliferation, migration, and invasion of A375 cells. (Chiu et al. 2022) found that the combination of curcumin and gefitinib not only enhanced the sensitivity of drug-resistant melanoma A375R cells to the drug but also reduced their viability and population. This combination therapy resulted in the reversal of tumor resistance. The relevant mechanism may involve modulation of the EGFR signaling pathway, which in turn affects the expression of Caspase-9 and Caspase-3 in A375R cells, thereby inducing apoptosis. Furthermore, photodynamic therapy, as a cancer treatment method, has been found to employ specific compounds like curcumin as photosensitizers in A375 cells. This approach sensitizes the otherwise drug-resistant A375 cells to the therapeutic agent, thereby combating drug resistance (Hosseinzadeh et al. 2021). Furthermore, studies have indicated that curcumin treatment in melanoma animal models can result in mild renal and hepatic inflammation and fibrosis, potentially exerting toxic effects on the organism. However, these symptoms have been shown to be mitigated by the co-administration of disulfiram, suggesting that the toxic effects of curcumin can be attenuated by other pharmaceuticals, thereby enhancing its antitumor efficacy (Fontes et al. 2022).

Some studies have found that DM-1, as a derivative of curcumin, can achieve antitumor effects by down-regulating the activities of matrix metalloproteinases (MMPs) that are associated with aggressiveness, such as MMP-2 and MMP-9. This, in turn, inhibits the invasiveness of *BRAF*-mutant melanomas, thereby exerting an antitumor effect (de Souza et al. 2020). In a separate study, the curcumin derivative EF24 was identified as an effective compound against drug-resistant melanoma (He et al. 2021). The mechanism of action involves the induction of apoptosis through two primary pathways: down-regulation of the unfolded protein response (UPR) signaling and inhibition of the NF-kB signaling pathway. These actions collectively contribute to the compound’s antitumor efficacy and its potential to overcome tumor resistance (He et al. 2021).

### Monomers of TCM of ginseng origin

Ginseng, recognized as warm in nature and characterized by a sweet and slightly bitter taste, is associated with the Heart, Lung, Spleen, and Kidney meridians in TCM. Its pharmacological effects include enhancing the body’s immune system, improving physical stamina, and promoting metabolic processes. Furthermore, ginseng exhibits antioxidant and antitumor activities. Ginsenosides, important active constituents of ginseng, are triterpenoid glycoside compounds. They can be classified into several groups, including protopanaxadiol group saponins (PPD saponins), protopanaxatriol saponins (PPT saponins), and oleanane-type saponins. To date, over 40 distinct ginsenosides have been identified (Xu et al. 2021). Among these, ginsenoside Rg3 has demonstrated anticancer effects across a spectrum of cancers. This includes gastric cancer (Wu et al. 2020), non-small cell lung cancer (Niu et al. 2023), and ovarian cancer (Zhao et al. 2021).

In a study, ginsenoside Rg3 was found to inhibit melanoma invasion and metastasis in the human malignant melanoma cell line A375.S2 by inducing apoptosis. This effect is achieved through the accumulation of Bax and the reduction of Bcl-2, thereby reversing melanoma’s drug resistance. The underlying mechanism may involve modulation of the MEK signaling pathway (Kim et al. 2019). Furthermore, ginsenoside Rg3 triggers apoptosis by facilitating Bax protein upregulation and Bcl-2 protein downregulation, thereby suppressing melanoma cell invasion and metastasis, aligning with the current research and targeted therapeutic approaches focused on apoptosis signaling pathways in contemporary oncology. In another study, ginsenoside Rg3 has been shown to induce cell cycle arrest in the S-phase of drug-resistant melanoma cells and reduce the expression of proliferating cell nuclear antigen (*PCNA*) (Meng et al. 2019). Furthermore, it down-regulates the expression of matrix metalloproteinases MMP-2 and MMP-9, thereby attenuating the migration of drug-resistant cells both *in vitro* and *in vivo*. The anti-tumor mechanism of action of ginsenoside Rg3 may be mediated through the inhibition of the ERK and AKT signaling pathways (Meng et al. 2019). Moreover, ginsenoside Rg3 has been demonstrated to reduce tumor vascularity and inhibit melanoma progression in xenograft animal models. Importantly, it was found to lack significant adverse effects on the animals and to offer protection against cisplatin-induced nephrotoxicity (Meng et al. 2019; Li et al. 2020; Zhang et al. 2021).

### Monomers of TCM of Astragalus membranaceus origin

Astragalus membranaceus, also known as Huang Jie, is characterized as warm in nature and sweet in taste. It is associated with the Lung and Spleen meridians in TCM. This herb is known for its various health benefits, including enhancing immune function, slowing the aging process, exhibiting antioxidant properties, and improving microcirculation. Astragalus polysaccharide (APS), one of the main active components of Astragalus membranaceus, exhibits a diverse spectrum of bioactivities. These include the regulation of the tumor immune microenvironment, anti-aging effects, intervention in type 2 diabetes, and antitumor properties (Liu et al. 2021). Research has demonstrated that APS possesses anti-tumor capabilities. APS facilitates the maturation of dendritic cells (DCs) by upregulating the expression of surface molecules CD80 and CD86. This process, in turn, activates cytotoxic T cells (CTLs), thereby enhancing the immune response against tumors (Chang et al. 2015).

Furthermore, APS can also act as a local mucosal adjuvant to inhibit melanoma metastasis. One study has shown that the combination of APS and an anti-PD-L1 antibody not only inhibited melanoma cells’ malignant infiltration of the lungs but also activated DCs through intranasal administration. This activation subsequently led to the engagement of natural killer (NK) and T cells, which together exerted the anti-melanoma effect (Hwang et al. 2021). Another study found that the combination of APS with cisplatin not only significantly slowed tumor growth in a mouse model induced by cisplatin-resistant melanoma cells, but also potentially down-regulated the expression of PD-L1 in these drug-resistant cells, contributing to the reversal of drug resistance. The underlying mechanism may involve the modulation of the PI3K/AKT signaling pathway (Gong et al. 2022).

In addition, it has been found that Honey-Roasted Astragalus Polysaccharide (HP-APS), obtained by mixing astragalus with honey, not only effectively inhibits the growth of melanoma cells *in vitro*, thereby playing a role in melanoma resistance, but also significantly enhances the expression of HSP70, CRT, MHC-1, CD86, CD80, and ATP release *in vivo*. This enhancement leads to an increase in the ratio of CD4+ to CD8+ T cells, boosts cellular immunity, and enhances anti-tumor effects (Sha et al. 2022).

In summary, warm herbal monomers can exert anti-melanoma effects through various mechanisms, including affecting the cell cycle, inducing apoptosis, modulating immune responses, and influencing signaling pathways. Consequently, warm herbal monomers may hold potential for the treatment of melanoma and other tumors.

## Hot-natured Chinese medicinal monomers

**Figure 5.**
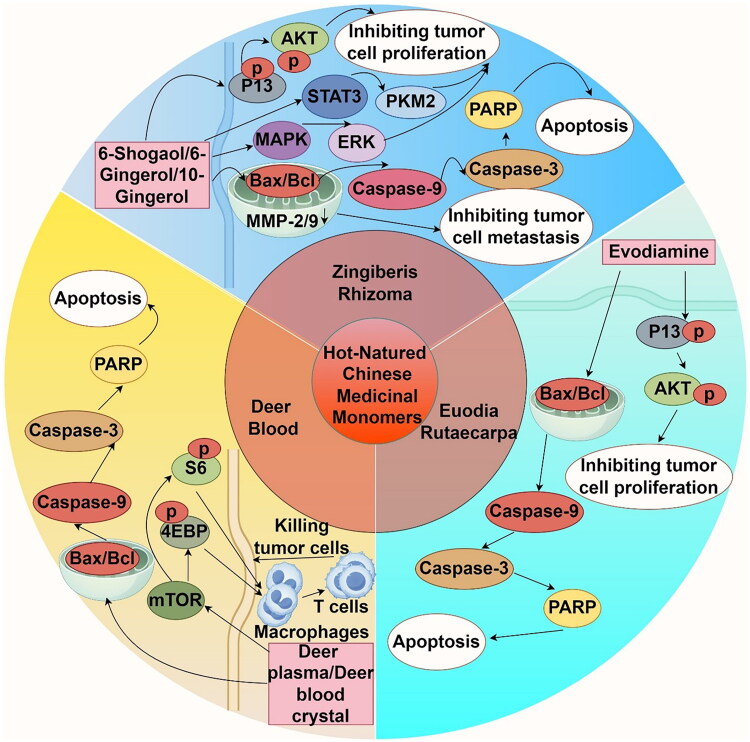
Mechanism diagram of the main actions of hot-natured Chinese medicinal monomers in melanoma (by Figdraw).

Herbs classified as ‘hot’ inTCM are typically pungent and are believed to correspond primarily with the heart, lung, and stomach meridians. They are known for their effects on promoting qi, nourishing the heart, warming the stomach, strengthening the spleen, and invigorating both blood circulation and menstrual flow. Monomers derived from hot herbs, including Zingiberis Rhizoma, Evodia Rutaecarpa, and Deer Blood, are resistant to the development of melanoma (Figure 5).

### Monomers of TCM of zingiberis rhizoma origin

Zingiberis rhizoma, characterized by its hot nature and pungent flavor, is associated with the heart, lung, stomach, spleen, and kidney meridians. It is a widely used and effective medicinal food in TCM, with its extracts known for their anti-inflammatory, antioxidant, and anti-tumor properties.

6-Shogaol, an alkylphenolic compound derived from Zingiberis rhizoma, exhibits a broad spectrum of pharmacological actions. 6-Shogaol exhibits anti-inflammatory and anti-tumor effects in both inflammatory conditions (Bischoff-Kont and Fürst 2021), such as rheumatoid arthritis (Li et al. 2023), and in various types of tumors (Yeh et al. 2019; Ajeigbe et al. 2022), including colorectal adenomas and renal cell carcinomas. Furthermore, it has been discovered that 6-shogaol diminishes the expression of MMP-2 and MMP-9 both *in vitro* and *in vivo*, thereby inhibiting tumor invasion and metastasis. The mechanism of action is likely related to the activation of the PI3K/AKT signaling pathway (Ma et al. 2022). Furthermore, research indicates that organometallic complexes centered on the natural product 6-gingerol may serve as innovative chemotherapeutic agents, specifically targeting the mitochondria and the endoplasmic reticulum-Golgi apparatus membrane in A2058 metastatic melanoma cells. These complexes enhance singlet oxygen generation, trigger caspase-dependent non-apoptotic cell death, and exhibit elevated cell-free antioxidant capacity (Sumithaa et al. 2022).

6-Gingerol, a precursor to 6-Shogaol extracted from Zingiberis rhizoma, has also demonstrated anti-tumor effects. In a study by Li et al. (2019), A375 cells were treated with varying concentrations of 6-gingerol. The results indicated that the compound inhibits melanoma cell proliferation in a concentration-dependent manner and modulates the expression levels of proteins such as Cleaved-Caspase-3, Cleaved-PARP, BIP, IRE1, and JNK, thereby exerting its anti-tumor activity.

10-Gingerol, a natural compound derived from zingiberis rhizoma, exhibits a range of biological activities, including anti-inflammatory, antioxidant, and antitumor effects. It has been identified as both a molecular target for cancer therapy (Huang et al. 2021) and a pharmacological enhancer (Liang et al. 2023). Studies have shown that 10-gingerol inhibits the viability and proliferation of A375 melanoma cells in a dose-dependent manner. Furthermore, it significantly suppresses the phosphorylation levels of proteins such as BRAF, MEK1/2, and P38, without affecting the phosphorylation of ERK1/2. These findings indicate that 10-gingerol may act as a potential inhibitor of melanoma, with its antitumor efficacy potentially linked to the inhibition of *BRAF* activation (Zhang et al. 2018).

### Monomers of TCM of Evodia rutaecarpa origin

Evodia rutaecarpa is hot in nature, pungent and bitter in taste, with small toxicity, and belongs to the liver, stomach, spleen and kidney meridians, in which evodia rutaecarpa alkaloid is a natural alkaloid extracted from the TCM Evodia rutaecarpa, and its pharmacological effects include anti-inflammatory, analgesic, inhibition of tumor cell activity and proliferation, and promotion of apoptosis of tumor cells.

A recent investigation has demonstrated that Evodiamine, functioning as a multi-targeted antiproliferative agent, selectively targets and suppresses the IRS4 as well as the downstream PI3K/AKT signaling cascade in vemurafenib-resistant melanoma cells. This action results in the arrest of cell cycle progression, the induction of apoptosis, the suppression of melanoma cell proliferation, and the augmentation of chemosensitivity when administered in conjunction with vemurafenib. (Guo et al. 2024). In another study, the combined effect of evodiamine and vemurafenib on A375 cells was investigated. The study revealed that this combination not only suppressed the proliferation of A375 cells but also triggered apoptosis. This was achieved by increasing the expression of pro-apoptotic proteins such as Bax and Caspase-3, while simultaneously decreasing the levels of phosphorylated Akt (p-Akt), phosphorylated NF-κB p65 (p-NF-κB-p65), and the anti-apoptotic protein Bcl-2. Furthermore, evodiamine was found to enhance the sensitivity of drug-resistant A375 cells to vemurafenib (Li et al. 2020).

However, evodiamine, while demonstrating antitumor efficacy in *in vivo* models, has been observed to induce weight loss and renal impairment in mice, suggesting a degree of systemic toxicity (Yang et al. 2024). Calcium overload has been identified as a contributing factor to the nephrotoxicity associated with evodiamine. Therefore, maintaining calcium homeostasis during tumor treatment is a critical strategy to mitigate toxicity. This underscores the importance of closely monitoring renal function and potential off-target effects in patients receiving evodiamine-containing therapies (Yang et al. 2024).

### Monomers of TCM of deer blood origin

In TCM, ingredients derived from the deer family, such as Cervus nippon (sika deer) and Cervus elaphus (red deer), are valued for their warming properties and their association with the liver and kidney meridians. These ingredients, characterized by a sweet and salty flavor, are traditionally used for their purported anti-tumor effects and their potential to counteract tumor resistance.

Deer blood extract, specifically deer blood crystals, has been shown to inhibit the proliferation of melanoma cells and induce their apoptosis. The proposed mechanism involves enhancing the phagocytosis of melanoma cells by macrophages and activating the mTOR signaling pathway. This activation leads to an upregulation of the phosphorylation levels of downstream targets such as 4EBP1 and S6K1. Consequently, this process increases the expression of inflammatory cytokines, including tumor necrosis factor-alpha (TNF-α), interleukin-6 (IL-6), and interleukin-1 beta (IL-1β), as well as the secretion of nitric oxide in macrophages, thereby exerting an anti-melanoma effect (Pan et al. 2020).

Furthermore, research has identified that deer plasma, an extract derived from the blood of cervids, in combination with cisplatin, exhibits synergistic anti-tumor effects in a melanoma mouse model. This combination therapy not only reverses the immunosuppressive state by elevating the ratio of CD8+ and CD69+ T cells but also modulates the expression levels of apoptosis-related proteins such as Bcl-XL and Bax, as well as the activation of Caspase-3. The underlying mechanism is hypothesized to involve CHOP-dependent apoptotic pathways (Wang et al. 2023).

In summary, medicinal monomers derived from hot nature herbs demonstrate potential anti-melanoma activities through diverse mechanisms, including the inhibition of melanoma cell proliferation, the induction of melanoma cell apoptosis, and the modulation of the tumor microenvironment. While these monomers have exhibited antitumor efficacy *in vitro*, concerns regarding their potential toxic side effects have been raised. Consequently, further research is essential to identify strategies for reducing toxicity and enhancing therapeutic effects. Additionally, it is crucial to establish the safety, efficacy, and precise mechanisms of action of these herbal monomers in clinical setting.

## Other properties of TCM monomers

**Figure 6.**
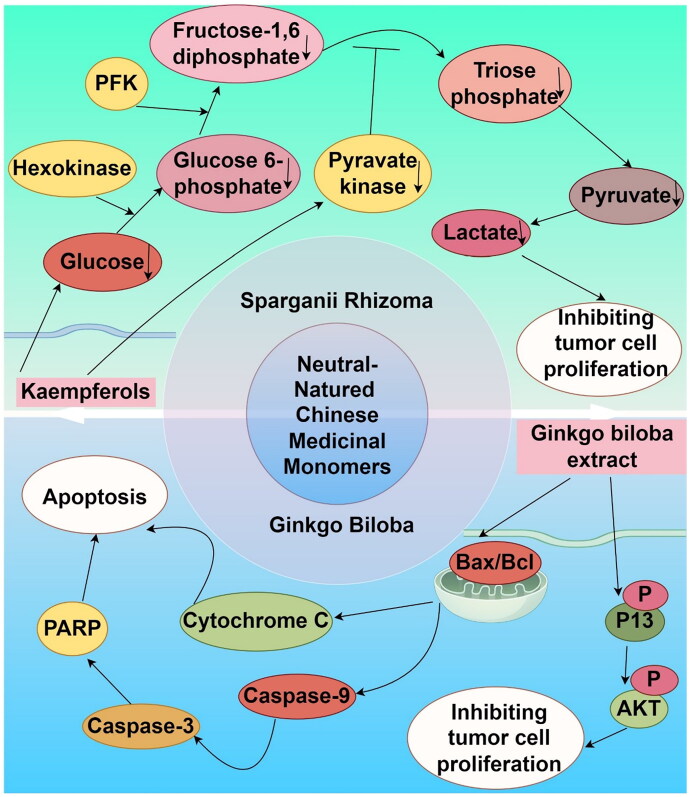
Mechanism diagram of the main actions of neutral-natured Chinese medicinal monomers in melanoma (by Figdraw).

In TCM, herbs with a neutral nature are characterized by their balanced properties and gentle effects, representing a distinct category beyond the typical classification of hot or cold herbs. These herbs are predominantly bitter and pungent in taste and are primarily associated with the liver, lung, and kidney meridians. They serve to nourish yin, strengthen the kidneys, clear heat, promote diuresis, and enhance the circulation of qi and blood. Medicinal monomers derived from such neutral herbs, including those from Sparganii Rhizoma (commonly known as San Leng) and Ginkgo Biloba, have demonstrated anti-inflammatory, antioxidant, and antitumor effects (Figure 6).

### Monomers of TCM of Sparganii Rhizoma origin

Sparganii Rhizoma, a plant from the Sparganiaceae family, is considered neutral in nature and bitter in taste in TCM. It is associated with the liver and spleen meridians. Extracts from Sparganii Rhizoma, particularly kaempferols, have been shown to possess multiple pharmacological properties, including antioxidant, anti-inflammatory, and antitumor activities. The proposed mechanism of action for these effects may involve the inhibition of the cellular glycolytic process, which could be a promising therapeutic strategy for melanoma treatment. Tumors that exhibit drug resistance, including NSCLC (Wang et al. 2022), non-Hodgkin lymphoma (NHL) (Xu et al. 2019), and melanoma, are known to display accelerated glucose metabolism. *BRAF* mutations in melanoma are associated with increased glycolysis. Inhibiting this metabolic pathway can delay the development of resistance to BRAF inhibitors, thereby enhancing therapeutic outcomes in melanoma with *BRAF* mutations (Brummer et al. 2019).

In a study by Liu et al. (2022), it was demonstrated that kaempferols can disrupt glycolysis in A375 melanoma cells, evidenced by reduced glucose uptake and diminished production of pyruvate, lactate, and ATP. Kaempferols also restrict the activity of pyruvate kinase, a key enzyme in glycolysis, culminating in the inhibition of cell proliferation. The proposed mechanism of action involves modulation of the expression of proteins implicated in glycolysis, including pyruvate dehydrogenase (PKM2), lactate dehydrogenase (LDHA), and the monocarboxylate transporter protein (MCT4), along with interference with glycolytic metabolism in tumor cells. Furthermore, kaempferol exerts antiproliferative effects on melanoma cells by inhibiting the glycolytic pathway, a mechanism that correlates with the modulation of metabolic pathways and represents a therapeutic target in contemporary oncology.

Furthermore, kaempferol has not exhibited significant toxic side effects, making it a promising candidate for therapeutic applications. Studies have demonstrated its efficacy in inhibiting the proliferation of melanoma cells in xenograft mouse models. Additionally, it has shown potential as an adjuvant to ameliorate liver and kidney injuries associated with colorectal cancer treatments. These findings suggest that kaempferol merits further investigation as a potential antitumor agent or supportive therapeutic (Qiang et al. 2021; Sharma et al. 2024).

### Monomers of TCM of Ginkgo biloba origin

Ginkgo biloba, a deciduous tree, is used in traditional medicine for its neutral properties and its sweet and bitter taste. It is associated with the lung and kidney meridians. Compounds extracted from Ginkgo biloba have been shown to possess a range of pharmacological effects, including antioxidant, antiviral, anti-inflammatory, and immunomodulatory activities (Fang et al. 2020).

A study conducted by has demonstrated that Ginkgo biloba leaf extract does not directly induce cell death in A375 melanoma cells (Chen et al. 2023). Instead, the extract exerts its antitumor effects by inhibiting the secretion of angiopoietin (ANG), thereby suppressing tumor cell invasion and angiogenesis induced by the tumor. The underlying mechanism is hypothesized to involve modulation of the integrin-linked kinase (ILK)/phosphatidylinositol-3-kinase (PI3K)/Akt signaling pathway. Extracts from Ginkgo biloba leaves have demonstrated the ability to induce apoptotic cell death in melanoma cells, characterized by DNA fragmentation, thereby exerting an anti-melanoma effect (Park and Kim 2015). Furthermore, these extracts activate the expression of key death-related proteins within the cytoplasm of melanoma cells, including p53, caspase-3, caspase-9, cytochrome c, and Bax, which are crucial for the induction of apoptosis. This mechanism suggests a potential therapeutic role for Ginkgo biloba leaf extracts in melanoma treatment (Park and Kim 2015).

In summary, herbal monomers with neutral properties, as classified in TCM, exhibit a range of anti-melanoma activities. These activities include the inhibition of melanoma cell proliferation, the induction of apoptosis, the promotion of healthy cell metabolism, and the modulation of immune system responses. Clinical applications of these neutral herbal monomers are extensive. With ongoing research, their therapeutic applications and potential are expected to expand significantly.

## Summary and outlook

The complexity of advanced melanoma and the diversity of therapeutic options necessitate personalized treatment plans that consider each patient’s stage and prognosis. Current mainstays of melanoma treatment include surgery, radiotherapy, immunotherapy, and targeted therapy. Although combination therapies and supportive care have improved outcomes, efficacy and long-term survival rates remain less than ideal. Chemotherapy remains a cornerstone of melanoma treatment. However, resistance, both innate and acquired, to chemotherapy is a primary cause of treatment failure. Therefore, further research is essential to investigate the potential of resistance-reversing agents, such as specific multi-target compounds that can address these resistance mechanisms.

We systematically review the anti-melanoma efficacy and pharmacological mechanisms of 25 traditional Chinese medicinal monomers with distinct properties, such as HHT, brusatol, mononoside, loganin, and curcumin, both *in vitro* and *in vivo* (Figure 7). These mechanisms include the inhibition of cell proliferation, induction of apoptosis, suppression of cell metastasis, and reversal of drug resistance. In this review, we outline the principal mechanisms by which traditional Chinese medicinal monomers with varying properties exert anti-melanoma effects: (i) HHT, brusatol, mononoside, loganin, and curcumin, EF24, ginsenoside Rg3, 6-gingerol, 10-gingerol, and evodiamine primarily inhibit melanoma by suppressing cell proliferation, promoting apoptosis, and modulating signaling pathways, (ii) 10-gingerol, β-elemene, curcumin and dihydroartemisinin sensitize melanoma cells to chemotherapy, mainly by regulating cell cycle progression, thereby enhancing anti-tumor activity, (iii) HHT, artemisinin, toadflax endolipids, curcumin, APS, evodiamine, and deer plasma potentially reverse drug resistance in melanoma by increasing the sensitivity of resistant cells to chemotherapeutic agents, (iv) baicalein, LXX-8250, 6-shogaol, and kaempferols engage in cellular metabolic pathways like glycolysis, thereby blocking cell proliferation, inducing apoptosis, and overcoming drug resistance to achieve anti-tumor effects, (v) baicalein, gambogic acid, curcumin, DM-1, ginsenoside Rg3, 6-shogaol, and evodiamine impede tumor progression by targeting proteins implicated in invasion and metastasis, (vi) baicalein, artemisinin, APS, deer blood crystals, deer plasma, and ginkgo biloba modulate the tumor microenvironment, inhibiting cell proliferation and increasing the efficacy of drugs against resistant cells. Despite their diverse chemical properties and distinct modes of action, herbal monomers share comparable mechanisms that contribute to their antitumor efficacy. By integrating these compounds, a synergistic approach can be developed to inhibit melanoma cell proliferation, promote apoptosis, and overcome drug resistance.

Figure 7.Structural formulas of some major Chinese medicinal monomers.

**Figure:**
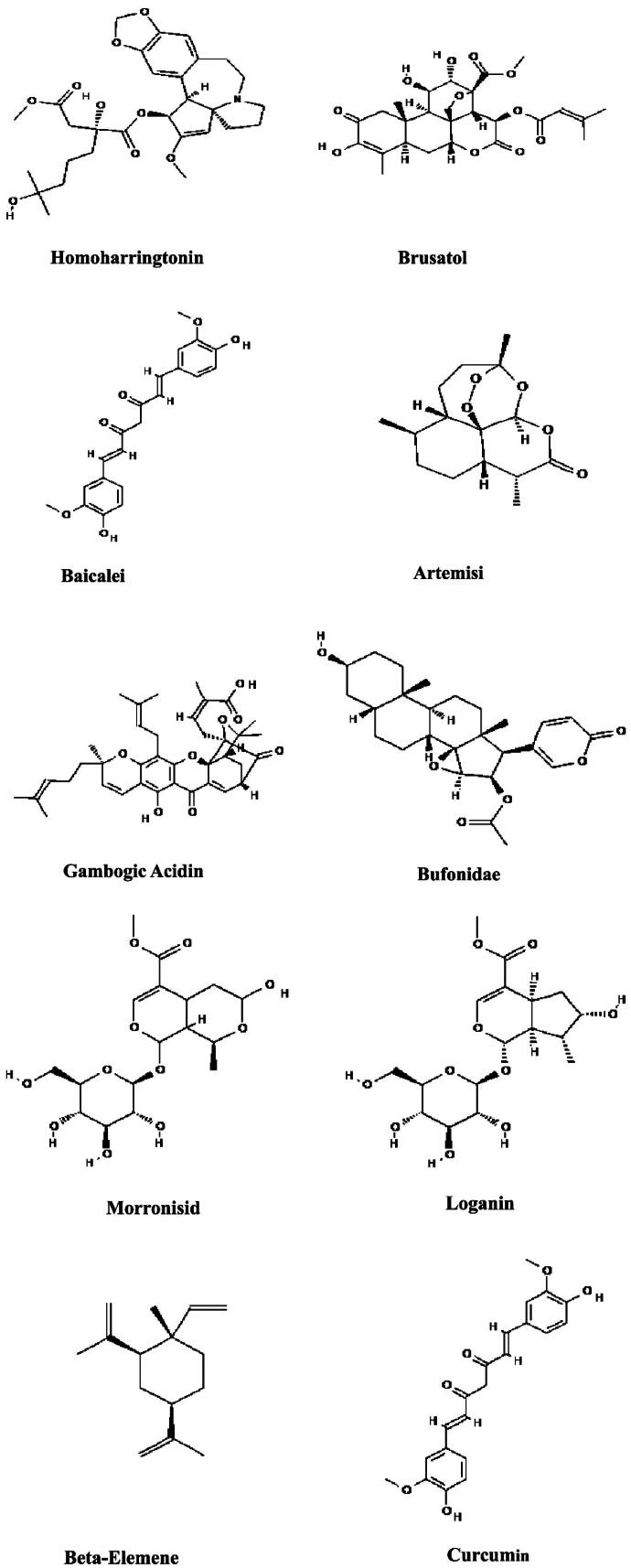

**Figure:**
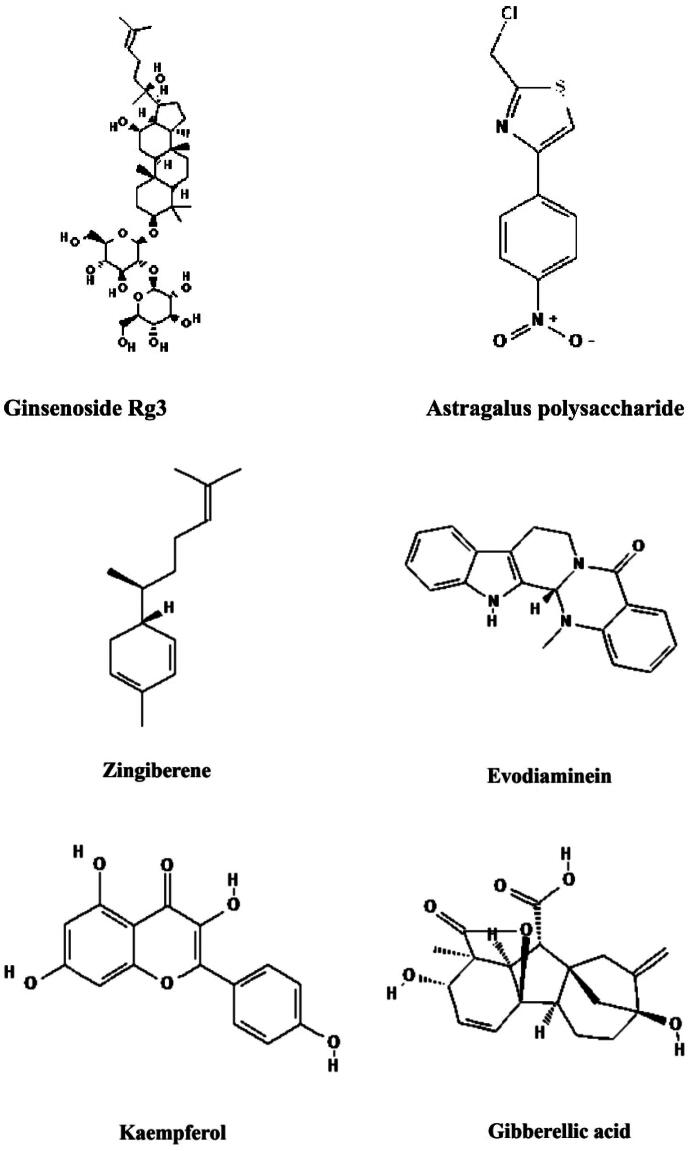

Owing to the intricate nature of melanoma development, the majority of herbal monomer research remains confined to *in vitro* experiments. Comparatively fewer studies have been conducted using *in vivo* models, and clinical trials are even more limited. Furthermore, current *in vitro* studies on herbal monomers have primarily focused on their impact at the protein expression level and on signaling pathways associated with melanoma proliferation, apoptosis, and drug resistance. There has been a notable lack of research into the transcriptional effects of these monomers on genes linked to melanoma phenotypes. High-throughput screening methods, which could elucidate these effects, have been rarely utilized in such studies. There is a clear need for further research into the potential of herbal monomers as both novel clinical therapies for melanoma and as components of combination treatments. These compounds hold promise for reducing drug resistance and improving patient outcomes.

## Data Availability

The data referenced in this study are primarily sourced from publicly accessible databases: PubMed and China National Knowledge Infrastructure (CNKI). Specific literature and datasets can be accessed as follows: PubMed: A DOI has been provided after each reference. China National Knowledge Infrastructure (CNKI): The Chinese literature has been translated into English, and links have been placed after the references. As this study did not generate new datasets or raw data, there are no additional data to share. All necessary data and analysis results are described in detail within the article and can be accessed through the aforementioned links.
